# Intracortical injection of endothelin-1 induces cortical infarcts in mice: effect of neuronal expression of an adenosine transporter

**DOI:** 10.1186/2040-7378-4-4

**Published:** 2012-03-12

**Authors:** Hanifi Soylu, Dali Zhang, Richard Buist, Melanie Martin, Benedict C Albensi, Fiona E Parkinson

**Affiliations:** 1 Departments of Pharmacology and Therapeutics, University of Manitoba, A404, 753 McDermot Avenue, Winnipeg, MB, Canada R3E 0 T6; 2 Departments of Radiology, University of Manitoba, Winnipeg, Canada; 3 Department of Physics, University of Winnipeg, Winnipeg, Canada; 4 Division of Neurodegenerative Disorders, St Boniface Hospital Research Centre, Winnipeg, MB, Canada

**Keywords:** Endothelin 1, Human Equilibrative Nucleoside Transporter 1, CD1, Mouse, Magnetic Resonance Imaging, Adenosine, Caffeine

## Abstract

**Background:**

Activation of adenosine A_1 _receptors has neuroprotective effects in animal stroke models. Adenosine levels are regulated by nucleoside transporters. In vitro studies showed that neuron-specific expression of human equilibrative nucleoside transporter 1 (hENT1) decreases extracellular adenosine levels and adenosine A_1 _receptor activity. In this study, we tested the effect of hENT1 expression on cortical infarct size following intracerebral injection of the vasoconstrictor endothelin-1 (ET-1) or saline.

**Methods:**

Mice underwent stereotaxic intracortical injection of ET-1 (1 μl; 400 pmol) or saline (1 μl). Some mice received the adenosine receptor antagonist caffeine (25 mg/kg, intraperitoneal) 30 minutes prior to ET-1. Perfusion and T_2_-weighted magnetic resonance imaging (MRI) were used to measure cerebral blood flow (CBF) and subsequent infarct size, respectively.

**Results:**

ET-1 reduced CBF at the injection site to 7.3 ± 1.3% (*n *= 12) in hENT1 transgenic (Tg) and 12.5 ± 2.0% (*n *= 13) in wild type (Wt) mice. At 48 hours following ET-1 injection, CBF was partially restored to 35.8 ± 4.5% in Tg and to 45.2 ± 6.3% in Wt mice; infarct sizes were significantly greater in Tg (9 ± 1.1 mm^3^) than Wt (5.4 ± 0.8 mm^3^) mice. Saline-treated Tg and Wt mice had modest decreases in CBF and infarcts were less than 1 mm^3^. For mice treated with caffeine, CBF values and infarct sizes were not significantly different between Tg and Wt mice.

**Conclusions:**

ET-1 produced greater ischemic injury in hENT1 Tg than in Wt mice. This genotype difference was not observed in mice that had received caffeine. These data indicate that hENT1 Tg mice have reduced ischemia-evoked increases in adenosine receptor activity compared to Wt mice.

## Background

Stroke is a leading cause of death and disabilities in developed countries. During stroke, a rapid depletion of ATP, dysregulation of ion channels and pumps, collapse of ion homeostasis, release of excitotoxic neurotransmitters, and intracellular calcium overload triggers a series of enzymatic cascades that result in neuronal death [1,2]. Unfortunately, with the exception of tissue plasminogen activator, there are as yet no clear evidence-based strategies for treatment [2].

Adenosine is a neuromodulator that acts through a family of metabotropic receptors. Activation of adenosine A_1 _receptors, which are widely distributed in brain, has neuroprotective effects. In contrast, activation of A_2A _receptors, which are most abundant in basal ganglia, can promote cell injury [3]. Adenosine can be formed extracellularly from catabolism of ATP by a cascade of ecto-nucleotidases [4], or can be formed intracellularly from ATP consumed in energy requiring processes followed by hydrolysis of AMP by cytosolic 5'-nucleotidases. Equilibrative nucleoside transporters (ENT) mediate cellular influx or efflux of adenosine and other nucleosides as dictated by their concentration gradients [5]. During pathophysiological events such as stroke or brain trauma, adenosine levels can increase up to 100-fold; the origin of this adenosine has been addressed *in vitro *using pharmacological tools but little information from *in vivo *studies is available [6,7].

Genetically altered mice can be used to investigate ischemic injury at the molecular level [8]. One method for producing a cerebral infarct in rodents is intracerebral injection of the vasoconstrictor peptide endothelin-1 (ET-1) [9-11]. Compared to other methods for producing stroke, such as distal middle cerebral artery occlusion or thromboembolic models, ET-1 induced focal lesions are relatively simple and reproducible [12]. Moreover, ET-1 is not directly neurotoxic but produces a temporary reduction in blood flow for several hours at the injection site [13,14].

In the present study ET-1 or saline was administered by intracortical injection to wild type (Wt) and transgenic (Tg) mice with neuron-specific expression of human ENT1 (hENT1) [15]. Local changes in cerebral blood flow and development of a cerebral infarct were monitored by magnetic resonance imaging (MRI) to test whether ENT1 over-expression affected stroke injury in a brain region expressing adenosine A_1 _receptors. The role of adenosine receptors in responses of Wt and Tg mice to ET-1 were examined with the antagonist caffeine.

## Methods

### Mice

CD1 mice with neuronal expression of hENT1 were generated as previously described [15]. To compare Tg to Wt mice, heterozygous Tg male mice were bred to Wt females and 8-10 week old (35-40 g) heterozygous Tg and Wt male littermates were used. All procedures and all outcome analyses were performed by individuals who were blinded to the genotype of the mice. All procedures with animals were approved by the University of Manitoba Animal Protocol Management and Review Committee.

### Surgical procedure

Mice were anesthetized initially with 5% isoflurane for induction then maintained with 1.5-2% isoflurane in 30% oxygen and 70% nitrous oxide. Rectal temperature was kept between 37-37.5°C through a thermal pad during the procedure and was maintained by a heating lamp during the recovery. Mice were placed in a stereotaxic apparatus. Following a midline incision to the scalp, a small burr hole was placed in the right hemisphere. A 5 μl Hamilton syringe was used to inject 1 μg ET-1 (Sigma-Aldrich Canada, Oakville, ON, Canada) (400 pmol in 1 μl of saline) or saline (1 μl) to the cortex in 5 minutes. One group of mice received caffeine (25 mg/kg) by intraperitoneal (i.p.) injection 30 minutes prior to intracortical injection of ET-1. The stereotaxic coordinates were determined from a mouse atlas [16] and were 1.0 mm anterior, and 1.0 mm lateral, to bregma and 1.2 mm below the pia. The needle was left *in situ *for 5 minutes to prevent back flow of ET-1 or saline before being slowly withdrawn. The hole was covered by bone wax and the incision was sutured.

### Magnetic resonance imaging

Mice were examined with perfusion-weighted MRI at 4 and 48 hours post-injection to confirm decreased cerebral blood flow (CBF). In addition, they were examined with T_2 _weighted MRI at 48 hours to determine infarct size. A Bruker Biospec 7 T/21 cm spectrometer (Bruker BioSpin, Karlsruhe, Germany) with a quadrature volume coil (National Research Council, Winnipeg, MB, Canada) 2.5 cm in diameter was used for MRI. Mice were anesthetized with 1.5 to 2% isoflurane in 30% oxygen and 70% nitrous oxide and placed in an animal holder with a thermal pad keeping body temperature at 37 to 37.5°C. The respiration rate was monitored using a pneumatic pillow connected to a respiratory monitor (Small Animal Instruments Inc., Stony Brook, NY, USA). Relative CBF was measured using an adiabatic spin labeling sequence with a 36 -echo HASTE readout following a 400 ms post tagging delay at 1 mm slice thickness and in-plane resolution of 234 μm. Slices were obtained at the plane of the injection site and at bregma for CBF measurement.

For T_2 _weighted images a multi-slice 8-echo RARE method with effective echo time TE = 80 ms and slice thickness 0.5 mm and 100 μm in-plane resolution was used. A total of 12 continuous slices were obtained with 1 mm inter-slice spacing in two 6-slice sets.

MRI analysis was performed using Marevisi 7.2 analysis software (National Research Council, Winnipeg, MB, Canada). The depth of ischemia was assessed by measuring relative blood flow within a 0.9 cm diameter circular area centered on the injection site. Those values were expressed as a percentage of pre-injection CBF values. Ischemia was defined as a decrease in CBF to less than 20% of pre-injection CBF [18]. Infarct size was obtained by measuring the area of hyperintense pixels in T_2 _weighted images, multiplying by the slice thickness and summing to obtain the infarct volume for each animal.

### Statistical analysis

The first study comparing outcomes of saline and ET-1 injections to Wt and Tg littermates was analyzed by two-way analysis of variance (ANOVA). In these ANOVAs, ipsilateral and contralateral CBF and ipsilateral infarct size were outcome variables and genotype (Wt or Tg) and drug (saline or ET-1) groups were treated as between-subject factors. For ipsilateral and contralateral CBF, assessments at 4 and 48 hours were examined for group differences. Follow-up pair-wise comparisons with Bonferroni adjustment of type I error were also conducted to examine differences across genotype and drug groups. The second study comparing outcomes of Wt and Tg littermates following i.p. injection of caffeine 30 minutes prior to intracortical injection of ET-1 was analyzed by two-way ANOVAs (CBF) or *t*-test (infarct size). No animals were excluded from analysis and no mortality occurred. All p-values were two-sided, and significance was set at a value of 0.05 for multiple comparisons. Statistical analyses were performed using SAS Version 9 (SAS Institute Inc, Cary, NC) or GraphPad Prism Version 5 (GraphPad Software Inc., LaJolla, CA).

## Results

### ET-1 and saline significantly decreased CBF at 4 hours post-injection

**Figure 1:**
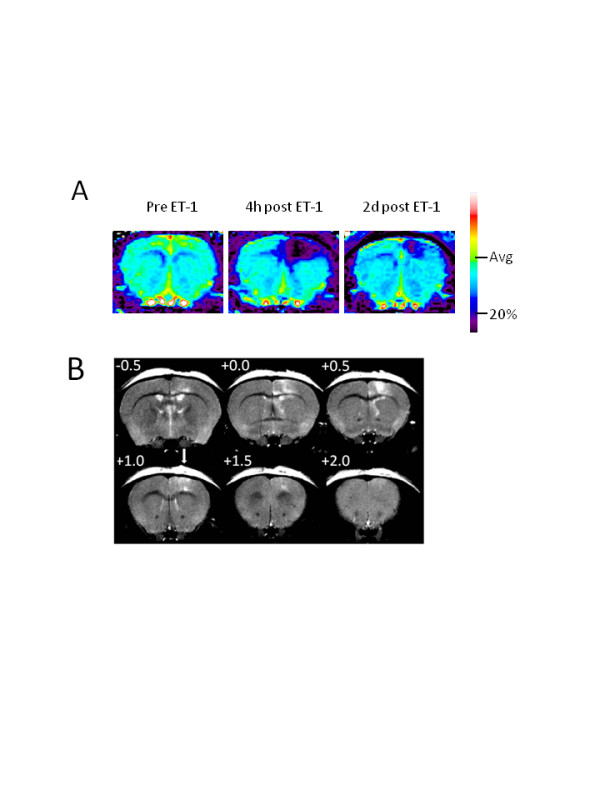
**Perfusion weighted and T_2 _weighted MR images after cortical injection of 1 μl ET-1**. ***A*. **Representative perfusion weighted MR images obtained from a Wt mouse before injection (pre; *left*) of ET-1 (400 pmol; 1 μl), and at 4 hours (4 h; *middle*) and 48 hours (2 d; *right*) post-ET-1 injection. Pseudocolor scale bar shows average cerebral blood flow (CBF) pre-injection (Avg) and ischemic threshold, set at 20% of pre-injection average (20%). ***B*. **Representative T_2 _weighted MR images obtained from a Wt mouse 48 hours post-injection of ET-1. In each panel, the number at the upper left shows the position relative to bregma. The arrow shows the injection site at +1.0 mm anterior to bregma.

**Figure 2:**
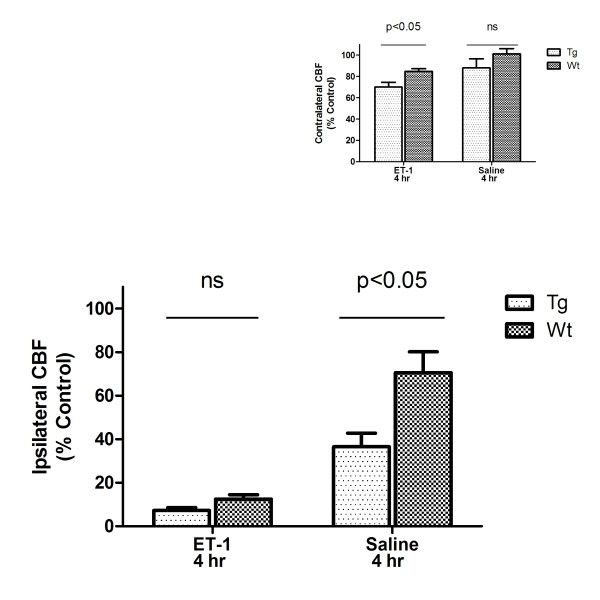
**Cerebral blood flow (CBF) is decreased 4 hours after intra-cortical injection of endothelin-1 (ET-1) or saline**. CBF was quantitated, relative to pre-injection set at 100%, in a 0.9 cm diameter circular region-of-interest centered on the injection site. Two-way ANOVA of ipsilateral CBF indicated a significant effect of genotype (F(1, 29) = 27.4, p < 0.01), a significant drug effect (F(1, 29) = 136.1, p < 0.001) and a significant genotype by drug interaction (F(1, 29) = 14.8, p < 0.01). Bonferroni post-tests indicated a significant difference (p < 0.05) between Wt and Tg mice injected with saline but not between Wt and Tg mice injected with ET-1. *Inset *ET-1, but not saline, injections were associated with decreased contralateral CBF. Two-way ANOVA of contralateral CBF at 4 hours also indicated a significant effect of genotype (F(1, 29) = 7.01, p < 0.05) and a significant drug effect (F(1, 29) = 10.6, p < 0.01). Bonferroni post-tests indicated that ET-1 produced a significantly greater decrease in contralateral CBF in Tg mice than in Wt littermates (p < 0.05). Data are means ± SEM, n = 12 Tg ET-1, 13 Wt ET-1, 4 Tg saline and 4 Wt saline.

Perfusion weighted MRI was performed at 4 and 48 hours following intra-cortical injections of ET-1 or saline and representative pseudocolor images are shown in Figure 1A. Compared to pre-injection values, ipsilateral CBF (± SEM) was decreased following injection of ET-1 or saline (Figure 2). Analysis of ipsilateral CBF at 4 hours indicated a significant effect of genotype (F(1,29) = 27.4, p < 0.01), a significant drug effect (F(1,29) = 126.1, p < 0.001) and a significant genotype by drug interaction (F(1,29) = 14.8, p < 0.01). Follow-up pair-wise comparisons indicated that ET-1 produced a greater decrease in CBF than saline, and a genotype difference was observed in mice injected with saline (p < 0.05) but not in mice injected with ET-1. Analysis of contralateral CBF at 4 hours also indicated a significant effect of genotype (F(1,29) = 7.01, p < 0.05) and a significant drug effect (F(1,29) = 10.6, p < 0.01) as ET-1 produced a greater decrease in contralateral CBF in hENT1 Tg mice than in Wt littermates (Figure 2, *inset*).

### CBF decreases persist 48 hours following injection of ET-1 but not saline

**Figure 3:**
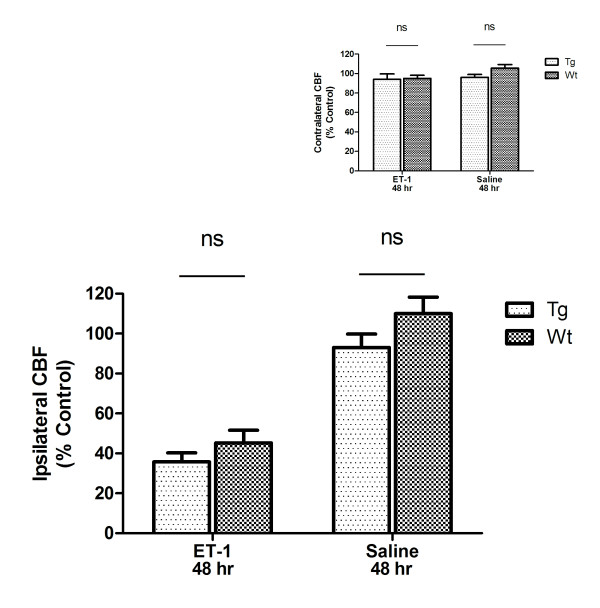
**Decreases in ipsilateral cerebral blood flow (CBF) persist for 48 hours after intra-cortical injection of endothelin-1 (ET-1)**. CBF was quantitated in a 0.9 cm diameter circular region-of-interest centered on the injection site. Two-way ANOVA revealed a statistically significant effect of drug (F (1, 29) = 50.9, p < 0.001) and Bonferroni post-tests revealed a significant effect of ET-1 for both Wt and Tg mice (p < 0.05). No significant differences between Tg and Wt were detected. *Inset *Contralateral CBF was restored to control levels 48 hours after injection of either saline or ET-1. Data are means ± SEM, n = 12 Tg ET-1, 13 Wt ET-1, 4 Tg saline and 4 Wt saline.

At 48 hours after injection of ET-1 the decrease in ipsilateral CBF was still evident whereas at 48 hours after injection of saline CBF was restored to pre-injection levels (Figure 3). Two-way ANOVA revealed a statistically significant effect of drug (F (1, 29) = 50.9, p < 0.001), with a significant difference between ET-1 and saline injected mice (p < 0.05). No significant effect of genotype was detected. The small decreases in contralateral CBF that were observed 4 hours after ET-1 injection were resolved by 48 hours (Figure 3, *inset*).

### Intra-cortical ET-1 injections produce a greater infarct size in hENT1 Tg than Wt mice

**Figure 4:**
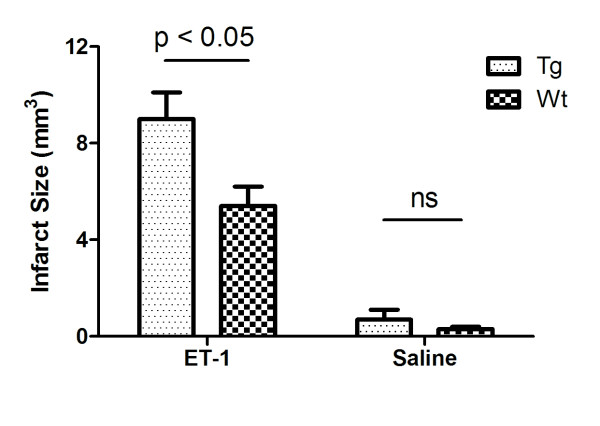
**Ischemic injury from ET-1 injection is greater in hENT1 Tg mice compared to Wt littermates**. Ischemic infarct size was determined from T_2 _weighted MRI at 48 hours post injection of either saline or ET-1. Two-way ANOVA indicated a significant drug effect (F(1, 27) = 22.3, p < 0.001). Bonferroni post-tests indicated no difference between Wt and hENT1 Tg mice injected with saline but a significantly larger infarct in hENT1 Tg, relative to Wt mice, following ET-1 injection (p < 0.05). Data are means ± SEM, n = 12 Tg ET-1, 13 Wt ET-1, 4 Tg saline and 4 Wt saline.

T2 weighted MRI was performed at 48 hours following intra-cortical injections of ET-1 or saline and representative images are shown in Figure 1B. Saline injections produced measurable infarcts (0.3 ± 0.1 mm^3 ^for Wt and 0.7 ± 0.4 mm^3 ^for hENT1 Tg) that were smaller than infarcts resulting from ET-1 injections (5.4 ± 0.8 mm^3 ^for Wt and 9.0 ± 1.1 mm^3 ^for hENT1 Tg) (Figure 4). Two-way ANOVA indicated a significant drug effect (F(1, 27) = 22.3, p < 0.001). Pair-wise comparisons indicated no difference between Wt and hENT1 Tg mice injected with saline but a significantly larger infarct in hENT1 Tg, relative to Wt mice, following ET-1 injection (p < 0.05).

### Caffeine abolishes genotype differences in response to ET-1 injections

**Figure 5:**
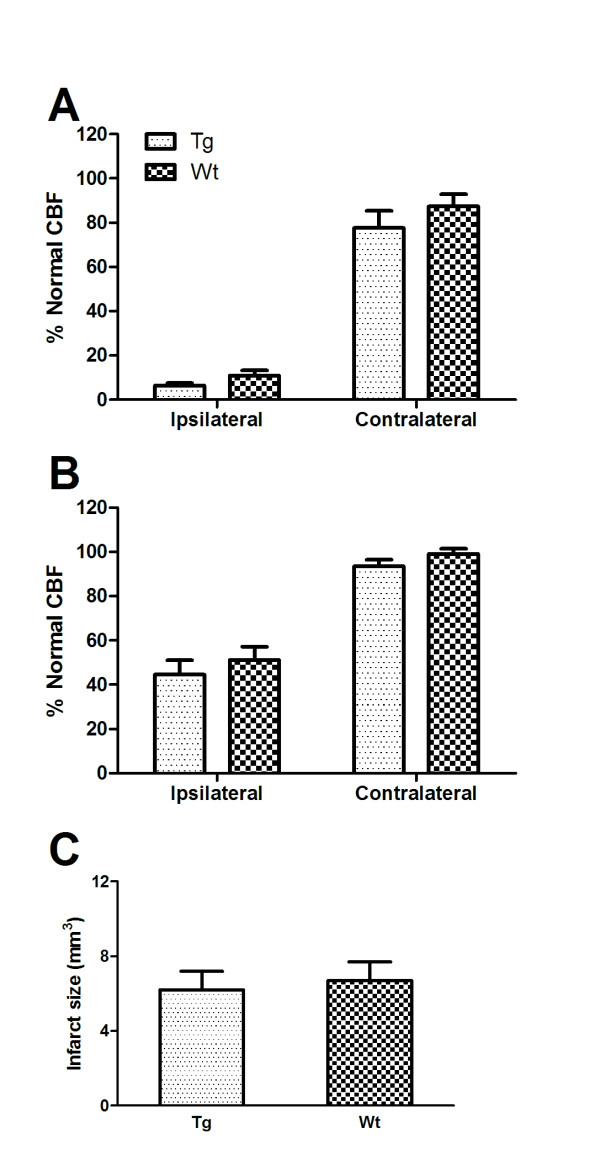
**Caffeine pre-treatment abolished the genotype difference in severity of ET-1 induced cerebral infarcts**. Mice were injected with the adenosine receptor antagonist caffeine (25 mg/kg, i.p.) 30 minutes prior to intracerebral injection of ET-1. ***A***. Ipsilateral CBF was decreased to less than 20% in both Tg and Wt mice; contralateral CBF was decreased by less than 20% at 4 hours post ET-1 injection. ***B*. **At 48 hours post ET-1 injection, partial recovery of ipsilateral CBF and full recovery of contralateral CBF was evident. ***C*. **Ischemic infarct size, determined 48 hours after ET-1 injection was similar in Tg and Wt mice. Data are means ± SEM; n = 10 Tg, 10 Wt mice.

The severe and long-lasting decrease in CBF following intracortical injection of ET-1 was also observed in mice pre-treated with caffeine. At 4 hours following ET-1 injection, ipsilateral CBF was reduced below the ischemic threshold of 20% in both Wt and Tg mice (Figure 5A). At 48 hours after ET-1 injection, ipsilateral CBF was restored to approximately 50% of pre-ischemic levels, in both genotypes (Figure 5B). At 4 hours after ET-1 injection, contralateral CBF was reduced to 77% and 87% in Tg and Wt mice, respectively, and was restored to 93% and 99%, respectively, by 48 hours following ET-1 injection. Ischemic infarct sizes, measured at 48 hours, were 6.2 ± 1 and 6.7 ± 1 mm^3^, and were not significantly different between Tg and Wt mice (Figure 5C).

## Discussion

The main finding of this study was that hENT1 Tg mice showed similar decreases in CBF, but had greater infarct sizes following ET-1 injection than Wt mice. Systemic pretreatment with the adenosine receptor antagonist caffeine abolished the genotype differences in infarct size, implicating differences in endogenous adenosine and adenosine receptor activity between Wt and hENT1 Tg mice.

ET-1 has been used previously to produce experimental strokes in rats [9,10,13] but mixed success has been reported with mice [8,11,17] leading some to conclude that ET-1 is not useful for mouse stroke models [8,12]. We found a significant CBF response to ET-1 injection that persisted for at least 48 hours ipsilaterally and led to cortical infarcts greater than 5 mm^3^. Using laser Doppler to measure CBF changes in mice after intracortical ET-1 injection, Wang et al. (2007) reported an 80% drop in CBF following 1 μg ET-1 injection, similar to the 90% drop in CBF at 4 hours that we report, and cortical infarcts of approximately 12 mm^3 ^[11]. Therefore, we conclude that ET-1 is an effective method for establishing a cortical infarct in mice.

We did observe one limitation to the use of ET-1. We found batch to batch variability in the stroke sizes produced by ET-1 injections. Thus, while the genotype difference in stroke size that we report here was also seen in pilot experiments, the average infarct sizes were different with each ET-1 preparation.

Previously, it has been reported that a decrease in CBF to 20% of control levels is required for stroke injury [18]. In this study, ET-1 administration produced an effect on CBF that equaled or surpassed this level of ischemia. The duration of the ischemic episode was not determined specifically, but was at least 4 hours. Using saline as a volume control, we found that mechanical or traumatic factors associated with injections produced decreases in CBF in ipsilateral cortex that persisted for 4 hours; however, these injections produced small infarcts that amounted to approximately 10% of the infarct sizes observed with injections of an equal volume of ET-1. From our data it appears that saline administration produced small cerebral infarcts without decreasing CBF below 20%. As CBF was determined using a circle of 0.9 cm diameter centered on the injection site, the small infarct sizes are likely associated with ischemic areas, of CBF below 20%, that are smaller than the 0.9 cm diameter circle. It is also possible that a more severe decrease in CBF occurred but resolved within 4 hours of saline injection.

Both saline and ET-1 produced measurable decreases in contralateral CBF; however, these decreases were modest and not of a magnitude associated with cerebral infarct. In studies using unilateral stroke models, it is sometimes assumed that the contralateral hemisphere is spared from CBF changes and that it can serve as an internal control [19]. However, contralateral cell death has been observed after middle cerebral and common carotid artery occlusion to ipsilateral hemisphere [19,20]. Furthermore, contralateral changes in hypoxia induced factor-1α, P-Akt, P-ERK1/2 and the proinflammatory cytokines IL-β, TNF-α and TNF-β have been observed in the absence of histopathological evidence for ischemic injury [21]. These results all indicate that a central autoregulatory system can affect CBF responses in brain regions beyond the immediate site of injury.

Adenosine is an important neuromodulator in many neurological events including ischemia, hypoxia and seizures [22]. During ischemic conditions, increases in adenosine levels of up to 100-fold have been measured [6,7]. This adenosine may be derived from neurons, astrocytes, or other cells that release ATP, which is then metabolized extracellularly to adenosine by ecto-nucleotidases [4]. Alternatively, adenosine can be released from cells via ENT-mediated efflux, a process that has been documented in neuronal cell cultures [23,24]. Other mechanisms of adenosine release, including vesicular release from neurons or astrocytes have been proposed [25-27]. In the present study, ET-1 was injected directly into a brain region that is rich in adenosine A_1 _receptors and expresses few A_2A _receptors. This injection produced a similar decrease in CBF in Wt and hENT1 Tg mice at the injection site yet the resulting cerebral infarct volume was significantly greater in hENT1 Tg mice relative to Wt. This indicates that hENT1 Tg mice had reduced endogenous neuroprotection or enhanced neurotoxicity, relative to Wt mice. Using hippocampal slice preparations, we recently reported that slices from hENT1 Tg mice show increased adenosine uptake and reduced adenosine receptor activity in response to hypoxic or ischemic conditions than slices from Wt mice [28], consistent with the results of the present study. Because the brain region targeted by ET-1 injections expresses A_1 _receptors at a much higher density than A_2A _receptors [29], it is likely that the effect of hENT1 expression to increase stroke injury is a direct consequence of reduced ischemia-induced increases in adenosine and decreased A_1 _receptor activation, relative to Wt mice [28]. A recent study using ENT1 null mice reported decreased ischemia-reperfusion injury to heart relative to Wt controls [30], which is consistent with the increased infact size in ENT1 over-expressing mice reported here.

Caffeine is a non-selective adenosine receptor antagonist with approximate K_i _values of 12 and 2.4 μM for A_1 _and A_2A _receptors [31]. Caffeine was used for this study because of its clinical significance as a commonly consumed psychoactive drug. Furthermore, caffeine has been used previously in Wt and hENT1 Tg mice and found to affect motor activity and ethanol-induced motor coordination [15,32]. The pharmacokinetics of caffeine has been examined in mice and a half-life of 25 minutes was reported [33]. Therefore, 30 minutes after caffeine treatment, at the time of ET-1 injection, caffeine levels are expected to still be in a range at which pharmacological effects have been observed in previous studies [15]. After caffeine administration, ET-1 no longer produced a significantly greater ischemic injury in Tg mice relative to Wt mice. These data further indicate that adenosine receptor activity is important for the difference in stroke injury size between Tg and Wt mice. The apparent lack of effect of caffeine in Tg mice may indicate that adenosine A_1 _receptor activity was minimal in the penumbral region in these mice, as a direct consequence of the increased neuronal uptake of adenosine mediated by functional hENT1.

## Conclusions

Cortical ET-1 injection is a relatively simple and reproducible technique for producing a cerebral infarct in mice. Following ET-1 injection, Tg mice expressing hENT1 in neurons showed a greater cerebral infarct size than Wt mice. Caffeine treatment prior to ET-1 injection abolished this genotype difference, implicating adenosine receptors in determining final infarct size. Our results can be explained by ENT mediated influx of adenosine into neurons and attenuated adenosine A_1 _receptor activity. This indicates that, during ischemia, neurons do not release adenosine via ENTs but actively salvage adenosine, which may be released by another mechanism, from another cell type, or formed extracellularly from released adenine nucleotides.

## Competing interests

The authors declare that they have no competing interests.

## Authors' contributions

HS and DZ participated in planning the experiments, performing the experiments, analyzing the MR images and writing the manuscript. RB and MM provided MRI expertise.

BAC participated in the design of the experiments.

FEP conceived of the study, participated in its design and coordination, and finalized the manuscript.

All authors read and approved the final manuscript.
